# Molecular Description of Eye Defects in the Zebrafish Pax6b Mutant, *sunrise*, Reveals a Pax6b-Dependent Genetic Network in the Developing Anterior Chamber

**DOI:** 10.1371/journal.pone.0117645

**Published:** 2015-02-18

**Authors:** Masanari Takamiya, Benjamin D. Weger, Simone Schindler, Tanja Beil, Lixin Yang, Olivier Armant, Marco Ferg, Günther Schlunck, Thomas Reinhard, Thomas Dickmeis, Sepand Rastegar, Uwe Strähle

**Affiliations:** 1 Institute of Toxicology and Genetics, Karlsruhe Institute of Technology (KIT), Postfach 3640, 76021 Karlsruhe, Germany; 2 Eye Center, Freiburg University Medical Center, Killianstr. 5, 79106 Freiburg, Germany; Purdue University, UNITED STATES

## Abstract

The cornea is a central component of the camera eye of vertebrates and even slight corneal disturbances severely affect vision. The transcription factor PAX6 is required for normal eye development, namely the proper separation of the lens from the developing cornea and the formation of the iris and anterior chamber. Human PAX6 mutations are associated with severe ocular disorders such as aniridia, Peters anomaly and chronic limbal stem cell insufficiency. To develop the zebrafish as a model for corneal disease, we first performed transcriptome and *in situ* expression analysis to identify marker genes to characterise the cornea in normal and pathological conditions. We show that, at 7 days post fertilisation (dpf), the zebrafish cornea expresses the majority of marker genes (67/84 tested genes) found also expressed in the cornea of juvenile and adult stages. We also characterised homozygous pax6b mutants. Mutant embryos have a thick cornea, iris hypoplasia, a shallow anterior chamber and a small lens. Ultrastructure analysis revealed a disrupted corneal endothelium. *pax6b* mutants show loss of corneal epithelial gene expression including regulatory genes (*sox3, tfap2a, foxc1a* and *pitx2*). In contrast, several genes (*pitx2, ctnnb2, dcn* and *fabp7a*) were ectopically expressed in the malformed corneal endothelium. Lack of *pax6b* function leads to severe disturbance of the corneal gene regulatory programme.

## Introduction

Transparency of the cornea is essential for normal vision. Corneal opacities can be caused by genetic disorders or corneal injuries. In early eye development, the ectoderm-derived lens separates from the corneal epithelium and mesenchymal neural crest cells invade to form the corneal stroma, endothelium, chamber angle and iris stroma. The anterior chamber is formed as the lens departs from the corneal structures. This process is affected in carriers of PAX6 mutations leading to a spectrum of clinical manifestations which can be sight-threatening and challenging to handle. Among them are dominantly inherited anterior chamber malformations such as aniridia (OMIM #106210) and Peters anomaly (OMIM #604229). Aniridia is characterised by incomplete iris morphology (iris hypoplasia) and often associated with other ocular abnormalities such as cataract, glaucoma, corneal opacification, vascularisation and limbal stem cell insufficiency leading to chronic ocular surface disease [1,2]. Peters anomaly patients present defects in the posterior layers of the cornea (stroma and endothelium) with corneal opacity, corneal adhesion of the lens and a high risk of glaucoma [3]. These patients often require corneal transplantation and cataract surgery in early infancy.

Animal models may serve to further characterise corneal PAX6-dependent signalling networks and allow for the development of improved treatment strategies. Mouse *small eye* (*Sey*) Pax6 homozygous mutants (*Sey/Sey*) die soon after birth, while heterozygous mice develop an abnormal anterior chamber with incomplete separation of the lens [4].

In zebrafish, two *PAX6* orthologues are expressed in the eye: *pax6a* and *pax6b* [5]. The *sunrise/pax6b* mutants carry a missense mutation in the homeobox that leads to reduced DNA binding and reporter gene activation [6]. However, in comparison to those in mammals, homozygous mutant embryos show milder eye defects and can be grown into adulthood with full fertility, suggesting functional complementation by the other co-orthologue *pax6a* [6]. There is evidence for the sub-functionalization of the two PAX6 orthologues at the level of synteny [6].

The anatomy of the zebrafish cornea is similar to that of other vertebrates. It lacks vasculature, is highly innervated and composed of three cellular layers: corneal epithelium, stroma, and endothelium [7]. This overall structure is already evident by 5 to 7 days post fertilisation (dpf). The innermost monolayer of cells, the corneal endothelium, is responsible for the transparency of the cornea [8], since it maintains a specific stromal hydration state. Surgical removal or ouabain poisoning of the zebrafish corneal endothelial layer leads to stromal swelling and opacity similar to the findings in human patients with corneal endothelial failure [9]. While the epithelial layer of the cornea is continuous with the epidermis, the corneal endothelium and the keratocytes of the stroma are derivatives of neural crest cells that migrate to the anterior chamber during early eye morphogenesis [10].

Although the mechanisms of zebrafish eye development and the use of zebrafish as model for human retinal disease have attracted a lot of attention [11,12,13,14], only few publications have addressed the cornea. Barely any specific molecular markers for *in situ* analysis of corneal gene expression have been described [15]. Here, we characterise the cornea transcriptome by both microarray and extensive *in situ* analysis of larval, juvenile and adult stages. We show that the cornea of a 7 dpf early larva already expresses many gene markers that we found expressed at 1-month juvenile and adult stages. We furthermore asked whether *pax6b* is required for cornea development. *pax6b* mutants show a smaller lens and a malformed cornea lacking a continuous uniform endothelial layer. Our data indicate that the expression of a large gene set is reduced in the corneal epithelium of *pax6b* mutants while a number of genes were ectopically expressed in the malformed endothelial layer in these animals. Thus, *pax6b* is required for normal gene expression in the cornea. In summary, we provide a comprehensive description of the molecular anatomy of the developing and mature zebrafish cornea.

## Results

### Transcriptional profiling of the zebrafish cornea

**Fig 1:**
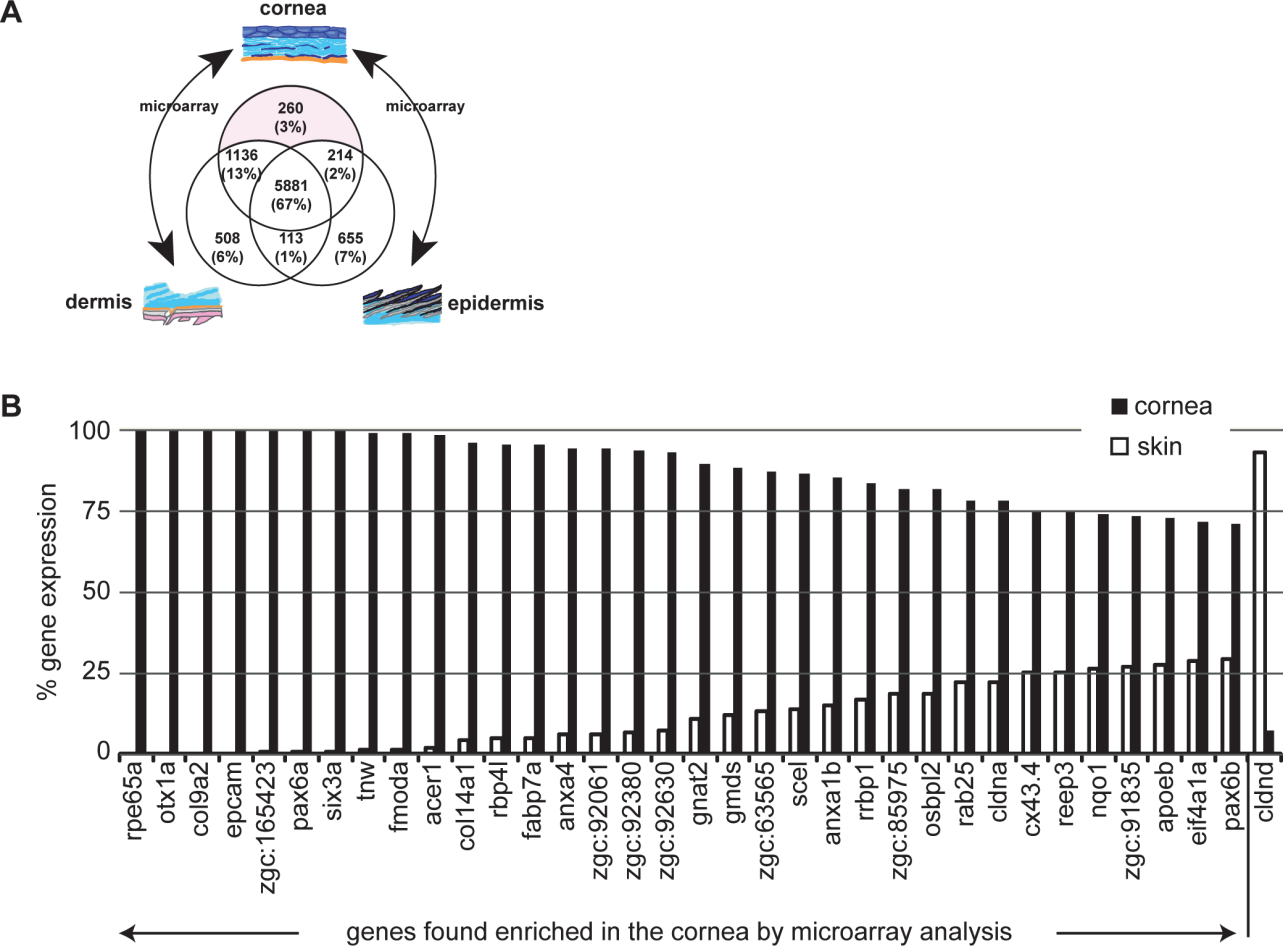
Identification of genes expressed in the adult zebrafish cornea. (A) Summary of the microarray results. In total 260 genes were found to be strongly expressed in the cornea relative to the dermis and epidermis. (B) RT-qPCR analysis of the gene expression in the cornea of adult zebrafish. Genes expressed differently between the two tissues in the microarray analysis (with fold change > 2 and adjusted *p*-value < 0.001) were selected and subjected to verification by RT-qPCR. Relative expression levels in the cornea (black columns) and the skin (white columns) are shown with the total expression level (cornea + skin) set to 100%. As an example for a gene enriched in the skin gene set, we chose *cldnd* (*claudin d*). All genes examined showed the correct direction of enrichment (three biological repeats, two tail unpaired *t*-test, *p*-adjusted <0.05) thus verifying our microarray results.

The layers of the cornea resemble the layers of the skin [16], and the corneal epithelial layer is derived from the ectoderm as is the epidermis of the skin. To identify genes that are expressed in the cornea of the adult zebrafish, we chose to compare the transcriptional profile of the cornea to that of the skin. The epithelium of the teleost skin is separated from the dermis by the mineralised scales [17,18,19]. We compared the transcriptome of the epidermis and the dermis of the adult zebrafish skin separately with the transcripts of the cornea. This approach was chosen for technical reasons as it is difficult to isolate the epidermis and dermis of the skin in a single specimen. All results are based on at least three independent biological repeats with dye-swap technical repeats. Data were normalised and processed as detailed in Materials and Methods. Among the 8767 genes represented commonly on the two different microarray designs used in this study, 2886 (32.9%) genes were differentially expressed (*p*<0.0007, adjusted *p*-value for multiple comparisons) (Fig. 1A; S1 Table). In the cornea preparations, the abundance of transcripts of 260 genes was significantly different (fold change >2.0, adjusted *p*-value <0.001) from the transcriptomes of dermis and epidermis (Fig. 1).

To validate the microarray data, we quantified the abundance of selected transcripts by RT-qPCR for genes whose transcripts were enriched in cornea- or dermis-derived total RNA. All the 89 genes re-analysed by RT-qPCR showed the predicted enrichment in either cornea or dermis (three biological repeats, two tail unpaired *t*-test, *p*-adjusted <0.05, Fig. 1B, Table 1), demonstrating that our microarray and data normalization strategy yielded robust differences in gene expression between skin and cornea. Four genes (*retinal pigment epithelium-specific protein 65a* (*rpe65a*), homeobox transcription factor *orthodenticle homolog 1a* (*otx1a*), collagen *col9a2* and *epithelial cell adhesion molecule* (*epcam*)) are exclusively expressed in the cornea with no detectable expression in the dermis (unique expression in the cornea, Fig. 1B, Table 1). The transcripts of other genes such as *tenascin W* (*tnw*, extracellular matrix glycoprotein), *paired box 6a* (*pax6a*) and *six homeobox 3a* (*six3a*) are several hundred fold enriched in the cornea, while the remaining genes show more moderate representations in the cornea sample using the RT-qPCR technique (Table 1). In summary, these results suggest that we achieved a good enrichment of genes expressed in the cornea by employing this microarray protocol of differential hybridization.

**Table 1:** RT-qPCR verification of the microarray data set.

Fold	ZFIN gene	name
unique^a^	*rpe65a*	*retinal pigment epithelium-specific protein 65a*
unique^a^	*otx1a*	*orthodenticle homolog 1a*
unique^a^	*col9a2*	*procollagen, type IX, alpha 2*
unique^a^	*epcam*	*epithelial cell adhesion molecule*
516.97	*zgc*:*165423*	*hypothetical protein LOC100101646*
470.81	*pax6a*	*paired box gene 6a*
385.80	*six3a*	*sine oculis homeobox homolog 3a*
119.16	*tnw*	*tenascin W*
96.94	*zgc*:*113456*	*fibromodulin-like*
69.14	*acer1*	*alkaline ceramidase 1*
24.46	*col14a1*	*collagen, type XIV, alpha 1*
19.44	*rbp4l*	*retinol binding protein 4, like*
19.38	*fabp7a*	*fatty acid binding protein 7, brain, a*
16.94	*anxa4*	*annexin A4*
16.61	*zgc*:*92061*	*hypothetical protein LOC436656*
14.56	*zgc*:*92380*	*hypothetical protein LOC445086*
13.24	*zgc*:*92630*	*hypothetical protein LOC436969*
8.45	*gnat2*	*guanine nucleotide binding protein (G protein), alpha transducing activity polypeptide 2*
7.27	*gmds*	*GDP-mannose 4*,*6-dehydratase*
6.79	*zgc*:*63565*	*hypothetical protein LOC386994*
6.19	*scel*	*sciellin*
5.86	*anxa1b*	*annexin A1b*
5.04	*rrbp1*	*ribosome binding protein 1 homolog (dog)*
4.41	*zgc*:*85975*	*homology to cytoskeleton-associated protein 4*
4.40	*osbpl2*	*oxysterol binding protein-like 2*
3.54	*rab25*	*RAB25, member RAS oncogene family*
3.51	*cldna*	*claudin a*
3.00	*cx43*.*4*	*connexin 43*.*4*
2.97	*reep3*	*receptor accessory protein 3*
2.79	*nqo1*	*NAD(P)H dehydrogenase, quinone 1*
2.76	*zgc*:*91835*	*drebrin-like*
2.66	*apoeb*	*apolipoprotein Eb*
2.49	*eif4a1a*	*eukaryotic translation initiation factor 4A, isoform 1A*
2.46	*pax6b*	*paired box gene 6b*
2.06	*chmp2bl*	*chromatin modifying protein 2B, like*
1.93	*capns1a*	*calpain, small subunit 1 a*

Genes expressed in the cornea were examined for gene expression by quantitative polymerase chain reaction following reverse transcription of total RNA (RT-qPCR) prepared from the adult zebrafish cornea and the dermis. Expression was normalized by using the level of expression of the *ef1a* gene as an internal control. The result is shown as the fold-change value of the expression level in the cornea in relation to the dermis. All genes presented in the table showed a direction of enrichment consistent with the microarray results (three biological repeats, two tail unpaired *t*-test, *p*-adjusted <0.05).

a) The label “unique” in the fold-change value indicates the absence of detectable transcripts in the dermis and hence exclusive expression in the cornea (*rpe65a, otx1a, col9a2* and *epcam*).

### Layer-specific expression in the cornea

**Fig 2:**
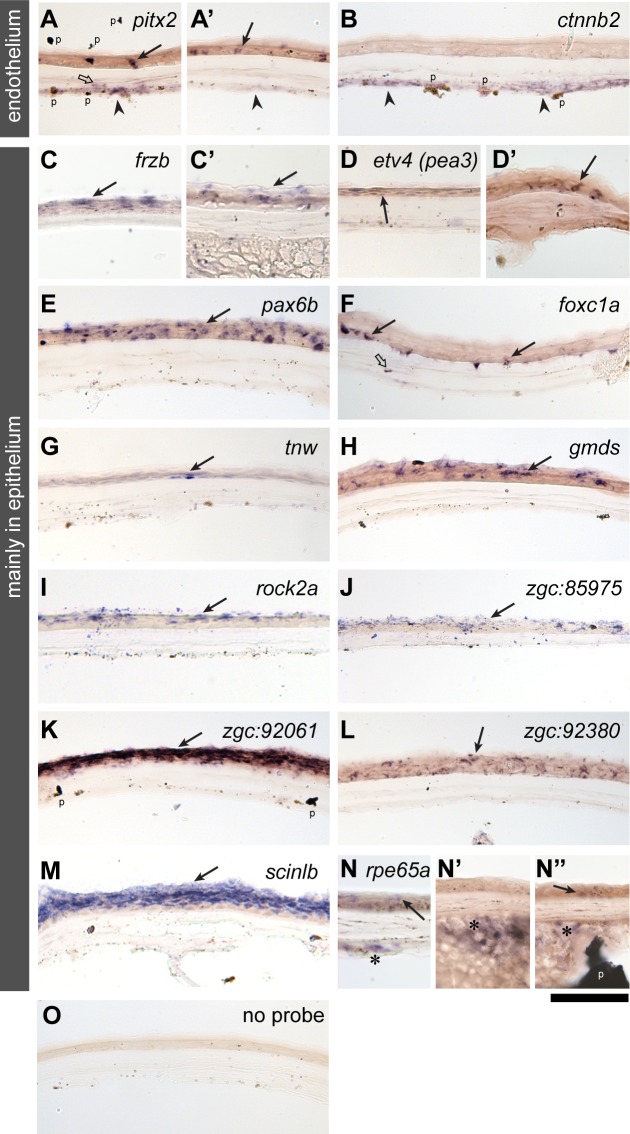
*In situ* mRNA expression analysis in the adult zebrafish cornea. Two major classes of expression patterns are shown for selected genes: genes mainly expressed in the corneal endothelium (*paired-like homeodomain 2* (A-A’) and *catenin, beta 2* (B)) and mainly in the corneal epithelium (*frizzled-related protein* (C), *ets variant 4* (previously known as *pea3*, D-D’), *paired box 6b* (E), *forkhead box C1a* (F), *tenascin W* (G), *GDP-mannose 4*,*6-dehydratase* (H), *rho-associated, coiled-coil containing protein kinase 2a* (I), *zgc*:*85975* (J), *zgc*:*92061* (K), *zgc*:*92380* (L), *scinderin like b* (M) and *retinal pigment epithelium-specific protein 65a* (N-N”). A control without probe is shown in the panel O. Filled arrows, open arrows, arrowheads and asterisks indicate expression in the corneal epithelial, stromal, endothelial layers and the annular ligament, respectively. The black “flakes” (indicated by p in the panels of A, B, K and N”) are derivatives of pigmentation unavoidably introduced during cryosectioning and its post-processing. Scale bar: 50 μm.

To assess in which layers of the cornea the genes enriched in the “cornea” set are expressed, we analysed the mRNA localisation of 32 genes by *in situ* hybridisation on cryostat sections of the adult eye (Fig. 2). This also allowed us to examine the expression patterns of the identified corneal genes relative to other ocular tissues. However, for technical reasons, the lens had to be removed prior to fixation and sectioning.

The *in situ* transcript distribution in the adult cornea can roughly be categorised into two patterns,—endothelial and epithelial expression, respectively. Two genes encoding Paired-like homeodomain 2 (Pitx2), a homeobox transcription factor whose human orthologue is implicated in Axenfeld-Rieger syndrome type 1 (OMIM #180500), and Catenin beta 2 (Ctnnb2), an adherens junction protein that also functions as a transducer of the canonical Wnt pathway, are detected mainly in the corneal endothelium (arrowheads in Fig. 2A-B), with less expression in the corneal epithelium (arrows in Fig. 2A-A’). The majority of genes examined *in situ* are expressed mainly in the corneal epithelium. These genes include signalling components, such as *frizzled-related protein* (*frzb*), encoding an extracellular canonical Wnt signalling regulator (arrows in Fig. 2C-C’; compare to no probe-negative control in Fig. 2O); *ets variant 4* (*etv4*, previously known as *pea3*; arrows in Fig. 2D-D’), encoding a transcription factor involved in the FGF signalling pathway; *GDP-mannose 4*,*6-dehydratase* (*gmds*, Fig. 2H), a rate-limiting enzyme in protein fucosylation required for full activation of Notch-Delta signalling [20]; and *rho-associated, coiled-coil containing protein kinase 2a* (*rock2a*, Fig. 2I), involved in the non-canonical Wnt (planar cell polarity) pathway [21]. Epithelial expression was also observed for the transcription factors *pax6b* (Fig. 2E) and *forkhead box C1a* (*foxc1a*, Fig. 2F), mutants of which cause anterior chamber dysgenesis in humans (PAX6 for Aniridia (AN #106210) and Peters anomaly (#604229); FOXC1 for Axenfeld-Rieger syndrome type 3 (RIEG3 #602482) and Iridogoniodysgenesis type 1 (IRID1 #601631)); an extracellular matrix protein *tenascin W* (*tnw*, Fig. 2G); an uncharacterised protein *zgc*:*85975* (Fig. 2J) with a homology to Cytoskeleton-Associated Protein 4 (CKAP4), a receptor for the Frizzled8-related antiproliferative factor [22] that is also implicated in steroid-induced glaucoma [23]; intermediate filaments, *zgc*:*92061* (*keratin 97*, Fig. 2K) and *zgc*:*92380* (Fig. 2L); zebrafish corneal crystallin *scinderin like b* (*scinlb*, Fig. 2M) [24]; all-trans-retinal-ester hydrolase with 11-*cis* retinol forming activity, *retinal pigment epithelium-specific protein 65a* (*rpe65a*, Fig. 2N-N”), which is involved in Leber congenital amaurosis 2 (OMIM #204100) [25] and Retinitis pigmentosa 20 (OMIM #613794) [26].

In summary, we have identified gene probes that differentially mark the corneal layers. Moreover, these *in situ* expression studies further verify the cornea gene set as a source of reliable molecular traits for further mechanistic studies.

### Many corneal genes are also expressed in the retina

**Fig 3:**
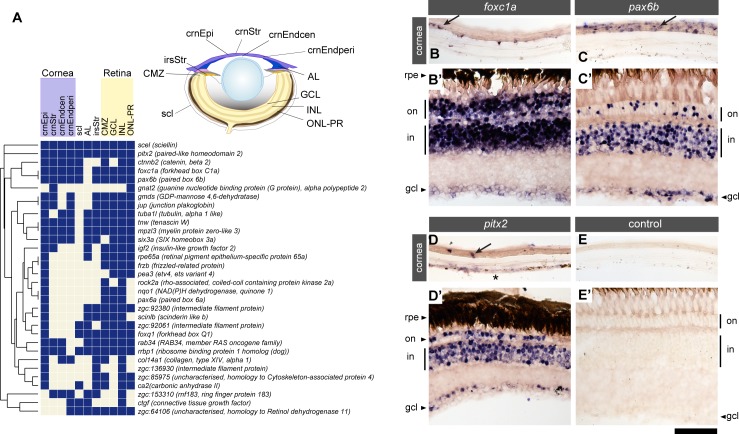
Genes expressed in the cornea are also expressed in the retina of the adult eye. (A) Analysis of genes and their expression in structures of the adult zebrafish eye. The 32 genes examined are listed along the vertical axis. The examined anatomical structures are shown in the schematic and listed on the horizontal axis. Anatomical structures examined are inner nuclear layer of the retina (INL), iris stroma (irsStr), outer nuclear layer and outer plexiform layer (ONL-PR), ganglion cell layer (GCL), ciliary marginal zone of the retina (CMZ), corneal epithelium (crnEpi), annular ligament (AL), sclera (scl), corneal stroma (crnStr), peripheral region of the corneal endothelium (crnEndperi) and central region of the corneal endothelium (crnEndcen). (B-E): Examples of genes expressed in the cornea and the retina. *in situ* gene expression patterns of *foxc1a* (B, B’), *pax6b* (C, C’) and *pitx2* (D, D’) are shown in the adult cornea (B-D) and retina (B’-D’), with tissue processed without antisense mRNA probe as a negative control (E, E’). Genes expressed in either the corneal epithelium (arrows) or the corneal endothelium (asterisk) are also expressed in the retina. rpe: retinal pigment epithelium; on: outer nuclear layer; in: inner nuclear layer; gcl: ganglion cell layer. Scale bar: 50 μm.

In the *in situ* hybridisation analysis, we realised that “cornea” genes are also expressed in the retina. Hence, we scored the patterns of expression in the eye systematically and subjected the sites of expression to cluster analysis (Fig. 3A). All of the examined “cornea” genes (*n* = 32) are also expressed in the retina. These include key transcription factors associated with congenital anterior chamber dysgenesis: *foxc1a* (Fig. 3B-B’), *pax6b* (Fig. 3C-C’), and *pitx2* (Fig. 3D-D’). Several “cornea” genes are also strongly expressed in the ciliary marginal zone (*scinlb, tuba1l, zgc*:*64106* and *NAD(P)H dehydrogenase, quinone 1 (nqo1*), a region that supports the lifelong growth of the retina in fish by generating new retinal neurons and glia cells [27,28]. These results suggest that the cornea and the retina share gene expression programmes at adult stages.

### The cornea of the 7 dpf larva already expresses the majority of genes of the adult cornea gene set

**Fig 4:**
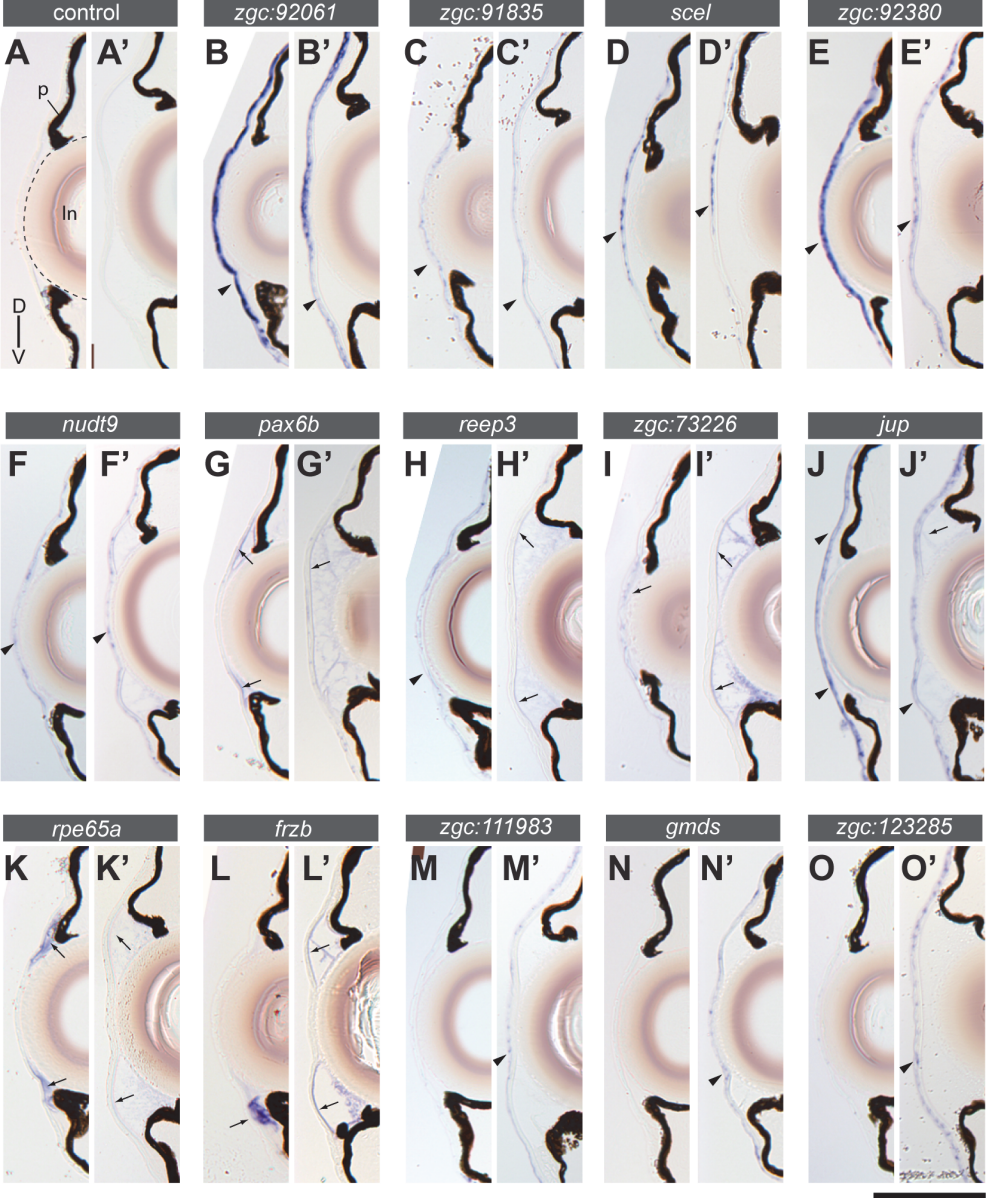
Comparison of gene expression in the cornea of wild type zebrafish at 7 dpf (A-O) and 1-month stage (A’-O’). Transverse sections with dorsal up. (A) Negative controls without probe showed no staining at both stages, except for background staining in the lens (ln, outlined by a stippled line). p: pigmentation in the iris and the retinal pigment epithelium. (B-K’) Examples of genes expressed at both 7 dpf and 1-month stage are shown. A subset of genes (B-F’) is mainly expressed in the corneal epithelium (arrowheads) and another subset (G-K’) shows expression in the corneal endothelium (arrows). (L-O’) Examples of genes that are mainly expressed at the 1-month stage. A subset of genes (M-O’) is expressed in the corneal epithelium (arrowheads) and another subset (L-L’) in the corneal endothelium (arrows). The gene symbols are indicated in the bar above each pair of sections. Scale bar: (A-O) 80 μm; (A’-O’) 100 μm.

**Fig 5:**
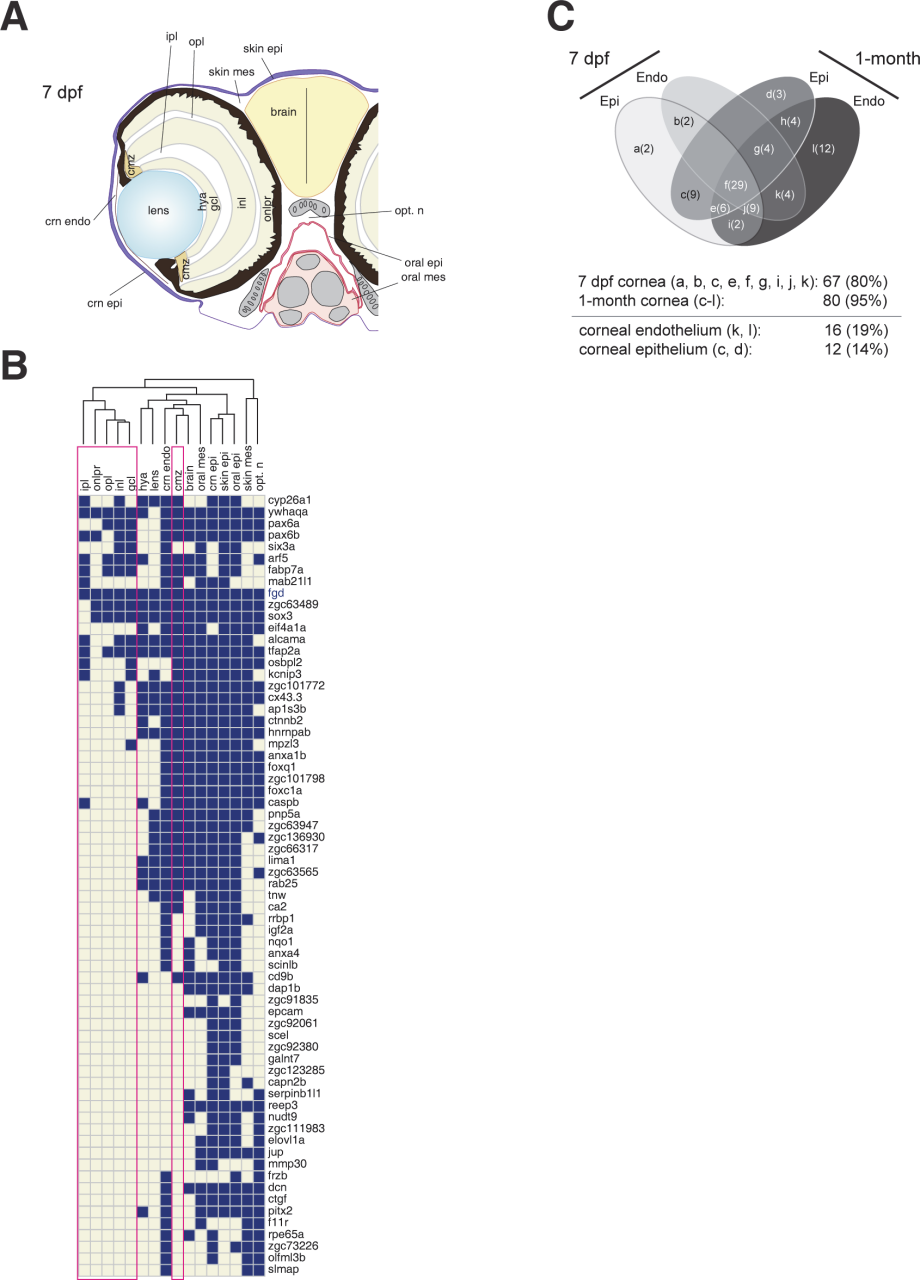
Expression of cornea genes in 7-dpf wildtype embryos in selected anatomical structures. (A) Schematic showing the 16 anatomical structures scored in the gene expression analysis: corneal epithelium (crn epi), corneal endothelium (crn endo), lens, ciliary marginal zone (cmz), hyaloid membrane (hya), ganglion cell layer (gcl), inner plexiform layer (ipl), inner nuclear layer (inl), outer plexiform layer (opl), outer nuclear layer-photo receptor (onlpr), skin mesenchyme (skin mes), skin epithelium (skin epi), brain, optic nerve fiber (opt. n), oral epithelium (oral epi) and oral mesenchyme (oral mes). (B) Cluster analysis of gene expression based on anatomical structures. The horizontal axis represents 16 anatomical structures and the vertical axis “cornea” genes. Among the examined genes (84 in total), only those with corneal expression are shown (67 genes). The columns surrounded by red rectangles indicate retinal expression (ipl, onlpr, opl, inl and gcl; cmz). Notice that 45% of the genes with corneal expression (30/67) are not expressed in the retina at 7 dpf, in contrast to the adult stage (100%, 32/32 examined, Fig. 3). (C) Four-way Venn’s diagram to summarize the corneal gene expression between 7 dpf and 1-month stages with respect to the corneal layers (epithelium (Epi) and endothelium (Endo)). The number of genes falling into each section (a-l) is shown in parenthesis. The 7-dpf and 1-month corneae express 80% (a, b, c, e, f, g, i, j and k; 67/84 genes) and 95% of the adult genes (c-l; 80/84 genes), respectively. The consistent corneal endothelium genes are *f11r, frzb, six3a, slmap, acer1, cd82, cldna, coch, col14a1, gdpd3, phgdh, ptgs2a, rock2a, zgc*:*153310* and *zgc*:*64106* (sections k,l; 16 genes). The corneal epithelium genes are *capn2b, elovl1a, jup, scel, zgc*:*111983, zgc*:*123285, zgc*:*91835, zgc*:*92061, zgc*:*92380, aclya, zgc*:*85975* (homology to CKAP4) and *gmds* (sections c, d; 12 genes).

The cornea of the zebrafish has developed the characteristic layered structure of the adult cornea by about 5 to 7 dpf [7,15]. We next wished to assess whether this early larval cornea has matured also with respect to expression of the “cornea” genes which we identified by expression screening of the adult cornea. We examined the *in situ* gene expression patterns of 84 “cornea” genes at two stages, 7 dpf and 1-month stage (Fig. 4; all image data are available in S1 Archives). To preserve the morphology and to include the lens for the analysis, we chose pre-embedding labelling (whole mount *in situ* hybridisation followed by embedding into epoxy resin) rather than *in situ* hybridisation on cryosections as we did for adult eyes. At 7 dpf, the majority of the adult corneal genes (80%, 67/84 examined; summarised in Fig. 5C) are already expressed in the cornea (Fig. 4B-L). This suggests that not only the overall morphology of the cornea has formed by 7 dpf, but the structures express already most of the genes characteristic for the adult cornea. However, the maturation is not totally completed at this stage as indicated by a significant number of genes (23%, 19/84 examined; *aclya, gmds, zgc*:*85975 (ckap4 homologue), gltpd1, cxcl14, sepw2b, idh1, rock2a, zgc*:*64106, ptgs2a, coch, cldna, gdpd3, crlf1a, acer1, cd82, col14a1, zgc*:*153310, phgdh*) that are not expressed at 7 dpf but started to be expressed at the 1-month stage (Fig. 4M-O’ and see also Fig. 5C). Besides differences in the onset of gene expression there are also differences in the pattern of expression. For example, the Wnt signalling regulator *frzb* [29] that is expressed in the entire corneal endothelium at 1 month of age (Fig. 4L’) is only expressed in a ventral patch at 7 dpf (Fig. 4L).

Next, we asked whether retina and the cornea share gene expression at 7 dpf. To address this issue, we scored the expression in the entire eye at 7 dpf (Fig. 5A-B). Due to low penetrance of the probes into deeper parts of the body of 1-month stage larvae, we excluded the 1 month stage from this analysis. Approximately half of the 67 genes with cornea expression are not expressed in the retina at 7 dpf (45%, 30/67 genes; Fig. 5B), in clear contrast to the adult stage (100% co-expression, 32/32 genes; Fig. 3A). This indicates that the retinal expression of around half of the corneal genes is acquired at stages later than 7 dpf. Interestingly, the expression in the ciliary marginal zone is the prevailing site where most of the corneal genes are expressed in the retina (36/37 genes) at 7 dpf (Fig. 5B).

### Zebrafish Pax6b mutants show an abnormal anterior chamber with severe corneal endothelium defects

**Fig 6:**
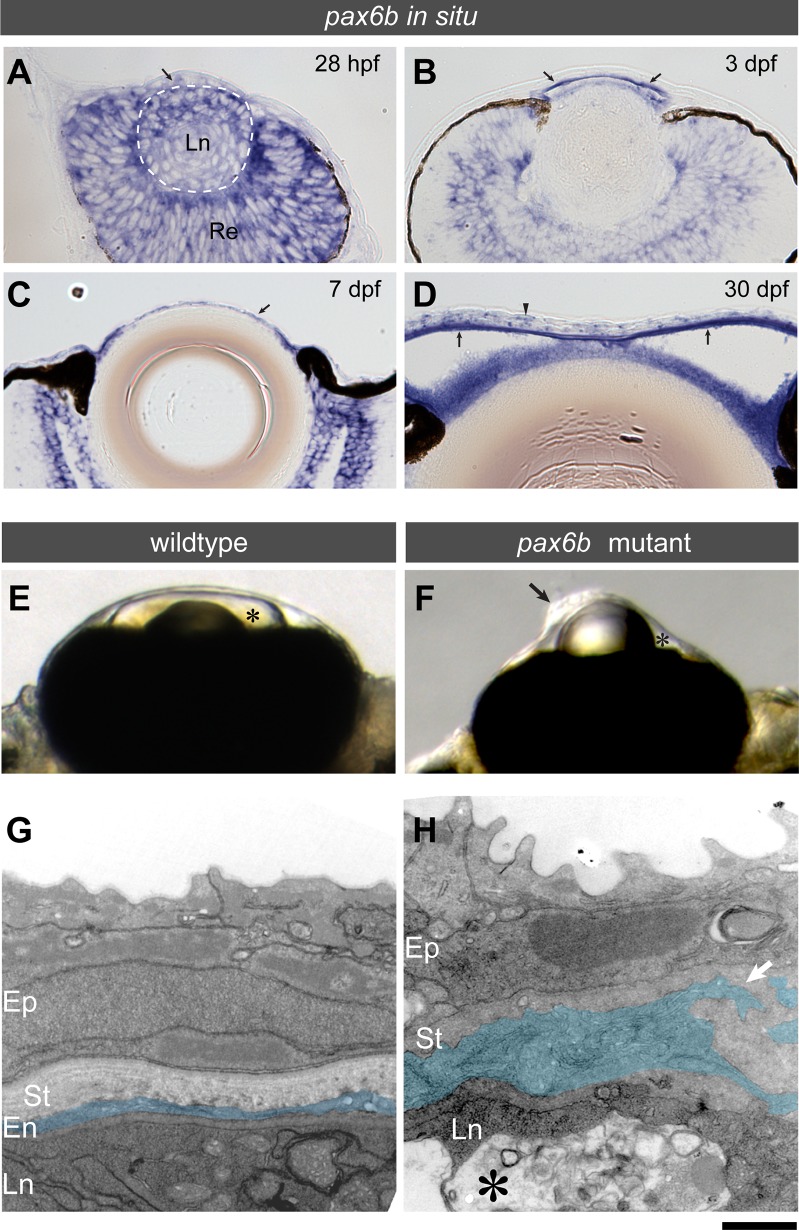
*pax6b* mutants (*also named sunrise*) present an abnormal anterior chamber with severe corneal endothelium defects. (A-D) *pax6b in situ* expression at 28 hpf (A), 3 dpf (B), 7 dpf (C) and 30 dpf (D). Transverse sections through the eye are shown with the ventral side left. (A) At 28 hpf, only a marginal level of *pax6b* transcripts is found in the ocular mesenchyme (arrow), well in contrast to high expression levels in the developing retina (Re) and the lens (Ln, stippled circle). (B-D) At 3 dpf, cells in the developing anterior chamber start to express *pax6b* (arrows in B), and its expression is maintained at 7 dpf (arrow in C) and 30 dpf (arrow in D). At 30 dpf, the corneal epithelium expresses *pax6b* transcripts (arrowhead, D) and its expression is maintained in the adult (Fig. 2E). (E-H) The anterior chamber phenotype of a *pax6b* mutant. (E-F) At 7 dpf, wildtype embryos have a well-formed anterior chamber (asterisk, E), while *pax6b* mutants show almost no anterior chamber (asterisk, F) and severely malformed corneal structures (black arrow, F) appear to be directly in contact with the lens. (G-H) Ultrastructure analysis of the cornea at 7 dpf. Wildtype embryos show the corneal endothelium (En, pseudo-coloured in blue) as consistent monolayer of cells. *pax6b* mutants lack a well formed corneal endothelium, and abnormal mesenchymal cells (pseudo-coloured in blue) with cellular protrusions (white arrow) were observed instead. Vacuolar deposits were present in the lens (asterisk). Ep: corneal epithelium, St: corneal stroma. Ln: lens. Scale bars: (A-D) 35 μm, (E-F) 93 μm and (G-H) 1.0 μm.

PAX6 mutations are the most common cause for corneal opacities present at birth [30]. Pax6b is expressed in the cornea of adult zebrafish (Fig. 2E). For these reasons, we analysed the Pax6b mutant *sunrise* [6]. The *sunrise* mutant allele carries a missense mutation L244P in the homeobox domain which leads to reduced DNA binding and reporter gene activation [6]. We analysed first *pax6b* mRNA expression in the eye in more detail during early embryonic development (Fig. 6A-D). At 28 hpf, when the anterior chamber and cornea have not differentiated yet (data not shown), only a marginal level of *pax6b* transcripts was detected in the ocular mesenchyme (arrow, Fig. 6A), in contrast to high expression levels in the developing retina and the lens. At 3 dpf, cells in the developing anterior chamber started to express higher levels of *pax6b* mRNA (arrows, Fig. 6B) and its expression was maintained at 7 dpf (arrow, Fig. 6C) and 30 dpf (arrow, Fig. 6D). At 30 dpf, the corneal epithelium expresses *pax6b* (arrowhead, Fig. 6D) and its expression was maintained in the adult (Fig. 2E).

The *pax6b* (*sunrise)* homozygous mutant embryos show abnormal anterior eye curvature recognizable as early as 19 hpf, when the lens placode starts delamination from the ocular surface ectoderm [31]. By 5–6 dpf, the lens is smaller in size in comparison to wildtype (Fig. 6E) and is positioned more externally with its centre coinciding with the margin of the optic cup (Fig. 6F). At the same time, the anterior chambers of *pax6b* mutant embryos are shallower than those of wildtype embryos (asterisks in Fig. 6E-F), accompanied with focal abnormal thickening of the cornea (arrow, Fig. 6F). Ultrastructure analysis suggests that the ocular mesenchyme (pseudo-coloured cells in blue, Fig. 6H) failed to properly differentiate into the corneal endothelium, as indicated by the presence of cellular protrusions (*n* = 3 embryos; white arrow, Fig. 6H) that are not observed in wildtype corneal endothelium (*n* = 3 embryos; pseudo-coloured in blue, Fig. 6G). Abnormal features are also observed in the corneal epithelium and the lens (black asterisk, Fig. 6H) with cytoplasmic vacuolation in *pax6b* mutant embryos, reminiscent of the mouse Pax6 *Sey* heterozygous lens [4]. Thus, zebrafish *pax6b* mutant embryos exhibit abnormal anterior chamber formation.

### Correct expression of cornea genes depends on *pax6b*

**Fig 7:**
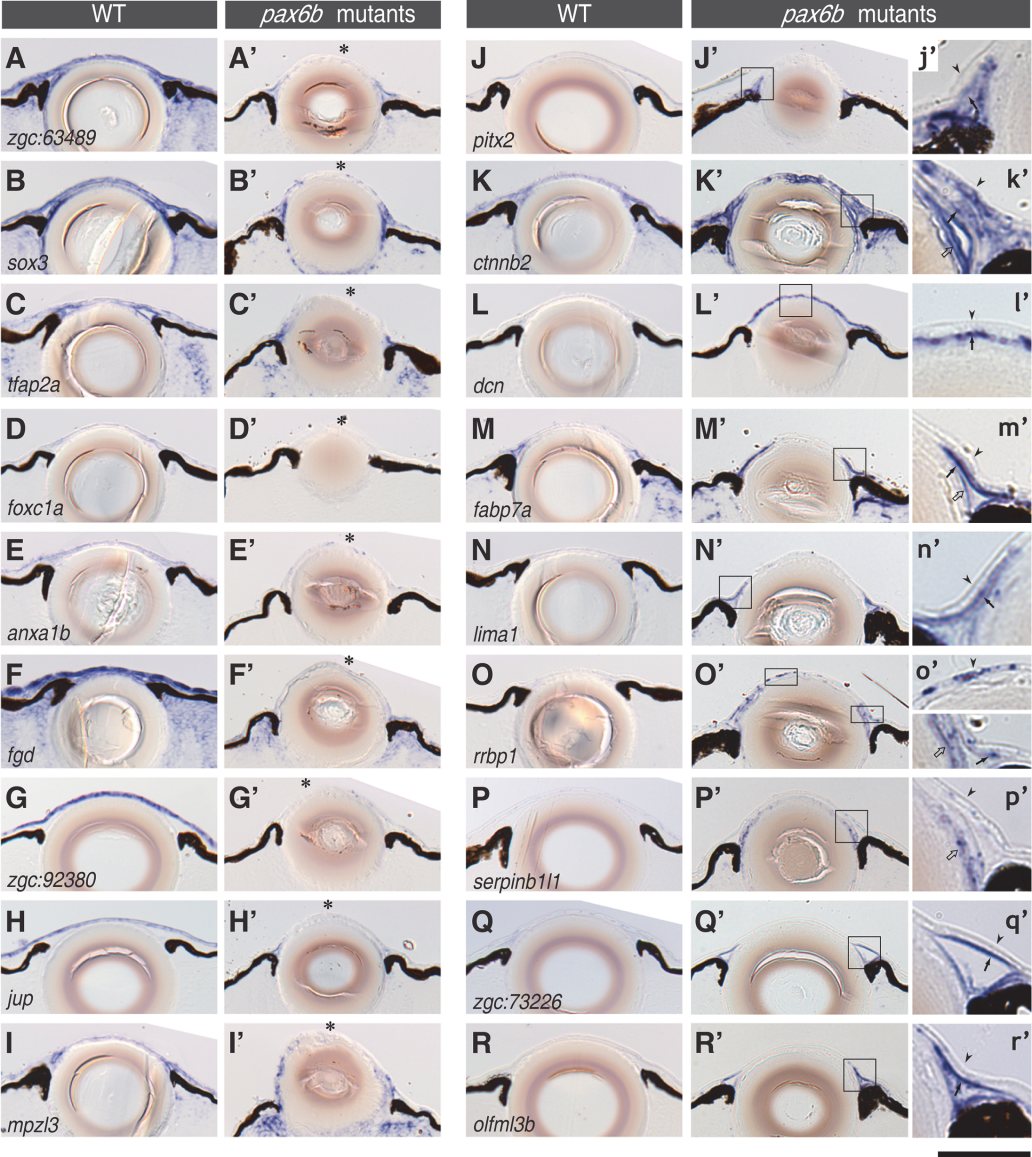
Gene expression in the cornea is severely affected in *pax6b* mutants. The distribution of the mRNA of cornea genes was compared at 7 dpf between wildtype (A-R) and *pax6b* mutant embryos (A’-R’). Each panel represents a transverse section through the eye and the lens. The gene transcripts analysed by *in situ* hybridization are *zgc*:*63489* (A-A’), *sox3* (B-B’), *tfap2a* (C-C’), *foxc1a* (D-D’), *anxa1b* (E-E’), *fgd* (F-F’), *zgc*:*92380* (G-G’), *jup* (H-H’), *mpzl3* (I-I’), *pitx2* (J-J’), *ctnnb2* (K-K’), *dcn* (L-L’), *fabp7a* (M-M’), *lima1* (N-N’), *rrbp1* (O-O’), *serpinb1l1* (P-P’), *zgc*:*73226* (Q-Q’) and *olfml3b* (R-R’). (A-I’) Examples for the loss of gene expression in the *pax6b* mutant (asterisks). (J-R’) Genes whose expression is ectopically induced in the *pax6b* mutant. The insets (j’-r’) represent magnified views of corresponding cornea regions (rectangular areas in J’-R’); arrowheads, filled arrows and open arrows indicate the corneal epithelium, the endothelium and the lens epithelium. The ectopic expression in *pax6b* mutants was mostly observed in the endothelial layer or in the iridocorneal angle. In contrast, the loss of gene expression in the *pax6b* mutant was mostly detected in the epithelial layer (asterisks in A-I). Scale bar: 64 μm (A-R’) and 20 μm (j’-r’).

Next, we compared the mRNA expression patterns of wildtype and *pax6b* mutant embryos using *in situ* hybridization with probes derived from 71 “cornea” genes as molecular traits. Each gene’s expression was compared in wildtype and *pax6b* mutant embryos at 7 dpf by staining wildtype and mutants in parallel under exactly the same conditions (Fig. 7). The results of the comparison showed that 59% of the corneal genes (42/71 analyzed genes) had altered expression patterns with reduced expression levels in the cornea of *pax6b* mutants (Fig. 7A-I’ and Table 2), while 28% genes (20/71 genes) were expressed in similar patterns as in the wildtype cornea, including *pax6b*. In contrast, 13% of the corneal marker genes (9/71 genes) were found ectopically expressed in the cornea/anterior chamber of *pax6b* mutants (Fig. 7J-R’). In total, expression of 72% of the corneal genes (*n* = 71 analysed genes) is affected in *pax6b* mutants. Interestingly, the effect of *pax6b* loss-of-function differs in the corneal epithelium and endothelium. The corneal epithelium showed loss of gene expression in the mutant (Fig. 7A-I’). In contrast, the malformed endothelium showed ectopic gene expression (Fig. 7J-R’). This is represented by the expression of *pitx2*, whose corneal epithelium expression is lost in the *pax6b* mutants, while ectopic expression is observed in the mutant endothelium (Fig. 7J-J’ and J’). Thus, a lack of *pax6b* function leads to major and complex gene expression changes in the cornea.

**Table 2:** Corneal gene expression in *sunrise/pax6b* mutants in comparison to wildtype embryos.

Gene expression in pax6b mutants	Gene name
Affected, down-regulated	alcama (activated leukocyte cell adhesion molecule a)
	anxa1b (annexin A1b)
	anxa4 (annexin A4)
	ap1s3b (adaptor-related protein complex 1, sigma 3 subunit, b)
	arf5 (ADP-ribosylation factor 5)
	ca2 (carbonic anhydrase II)
	capn2b (calpain 2, (m/II) large subunit b)
	cd9b (CD9 antigen, b)
	cx43.3 (connexin 43)
	dap1b (death associated protein 1b)
	elovl1a (elongation of very long chain fatty acids (FEN1/Elo2, SUR4/Elo3, yeast)-like 1a)
	epcam (epithelial cell adhesion molecule)
	f11r (F11 receptor)
	fgd (faciogenital dysplasia)
	foxc1a (forkhead box C1a)
	foxq1 (forkhead box Q1)
	galnt7 (UDP-N-acetyl-alpha-D-galactosamine: polypeptide N-acetylgalactosaminyltransferase 7)
	gpc1b (glypican 1b; zgc:63947)
	hnrnpab (heterogeneous nuclear ribonucleoprotein A/B)
	igf2a (insulin-like growth factor 2a)
	jup (junction plakoglobin)
	kcnip3 (Kv channel interacting protein 3, calsenilin)
	mboat1 (membrane bound O-acyltransferase domain containing 1; zgc:101798)
	mpzl3 (myelin protein zero-like 3)
	nqo1 (NAD(P)H dehydrogenase, quinone 1)
	nudt9 (nudix (nucleoside diphosphate linked moiety X)-type motif 9)
	osbpl2 (oxysterol binding protein-like 2)
	padi2 (peptidyl arginine deiminase, type II; zgc:66317)
	pnp5a (purine nucleoside phosphorylase 5a)
	rab11al (RAB11a, member RAS oncogene family, like; zgc:63565)
	rab25 (RAB25, member RAS oncogene family)
	sox3 (SRY-box containing gene 3)
	tfap2a (transcription factor AP-2 alpha)
	tnw (tenascin W)
	ywhaqa (tyrosine 3-monooxygenase/tryptophan 5-monooxygenase activation protein, theta polypeptide a)
	zgc:101772
	zgc:123285
	zgc:136930
	zgc:63489
	zgc:91835
	zgc:92061
	zgc:92380
Affected, up-regulated	ctnnb2 (catenin, beta 2)
	dcn (decorin)
	fabp7a (fatty acid binding protein 7, brain, a)
	lima1 (LIM domain and actin binding 1)
	olfml3b (olfactomedin-like 3b)
	pitx2 (paired-like homeodomain transcription factor 2)
	rrbp1 (ribosome binding protein 1 homolog (dog))
	serpinb1l1 (serpin peptidase inhibitor, clade B (ovalbumin), member 1, like 1)
	zgc:73226
Not affected	caspb (caspase b)
	ctgf (connective tissue growth factor)
	cyp26a1 (cytochrome P450, subfamily XXVIA, polypeptide 1)
	eif4a1a (eukaryotic translation initiation factor 4A, isoform 1A)
	frzb (frizzled-related protein)
	homer3 (homer homolog 3 (Drosophila); zgc:55557)
	mab21l1 (mab-21-like 1)
	mmp30 (matrix metallopeptidase 30)
	pax6a (paired box gene 6a)
	pax6b (paired box gene 6b)
	phgdh (phosphoglycerate dehydrogenase)
	psat1 (phosphoserine aminotransferase 1)
	rab34 (RAB34, member RAS oncogene family)
	reep3 (receptor accessory protein 3)
	rpe65a (retinal pigment epithelium-specific protein 65a)
	scel (sciellin)
	scinlb (scinderin like b)
	six3a (sine oculis homeobox homolog 3a)
	slmap (sarcolemma associated protein)
	zgc:111983

Sections of wildtype and *sunrise* homozygous mutant embryos at 7 dpf were subjected to *in situ* gene expression analysis with 71 cornea gene probes. Gene expression in the mutant was compared with that of wildtype embryos. The results were grouped into three cases: 1) affected, down-regulated (42 genes), 2) affected, up-regulated or ectopically expressed (9 genes) and 3) not affected (20 genes).

## Discussion

We characterised the gene expression in the zebrafish cornea by transcriptome and *in situ* expression analysis. In total, 260 expressed genes were identified in the adult cornea. The majority of the genes tested (80%, *n* = 84 genes tested) were already expressed in the cornea at 7 dpf, suggesting that the early larvae have already formed most of the adult corneal features. Furthermore, correct differentiation of the cornea requires the *pax6b* gene. *pax6b* mutants show complex changes in gene expression patterns; while expression of many genes in the epidermis is lost, expression of a smaller subset of genes is ectopically expressed in the malformed endothelial layer.

### The identified cornea genes comprise gene ontologies related to human corneal diseases

By differentially screening the cornea transcriptome against that of the skin (dermis, epidermis), we identified 260 genes whose transcripts are enriched in the adult zebrafish cornea as verified by RT-qPCR and *in situ* expression analysis. Among other gene ontologies, the “cornea” gene set comprises transcriptional regulators (*pax6a/b, pitx2, sp1* (*sp1 transcription factor*), *klf4* (*Kruppel-like factor 4)* and *foxc1a*), which were known from the mammalian literature to be crucial for cornea development and maintenance [32,33,34,35,36]. Several identified genes have human homologues, which are directly or indirectly involved in corneal dystrophies such as *coch* (*coagulation factor C homolog, cochlin* [37], *dcn* (*decorin*) [38], *cx43*.*4* [39], *clu* (*clusterin*) [40], *foxc1a* (Axenfeld-Rieger syndrome type 3 (RIEG3 #602482) and Iridogoniodysgenesis type 1 (IRID1 #601631)), *kera* (*keratocan*; posterior amorphous dystrophy [41]) and *tgfbi* (*transforming growth factor, beta-induced*; lattice corneal dystrophy [42]). Other genes were implicated in wound healing of the cornea (*ctgf, connective tissue growth factor* [43]; *cyp26a1, cytochrome P450, family 26, subfamily A, polypeptide 1* [44]; *f11r, F11 receptor* or *junctional adhesion molecule-A* [45]) or where identified as potential therapeutic targets (*arf1, ADP-ribosylation factor 1* [46]; *ccl1, chemokine (C-C motif) ligand 27a* [47]). Taken together, the set of 260 genes reported here represents a powerful tool to study development and pathological alterations of the zebrafish cornea. In addition, our gene ontology analysis strongly suggests that investigation of these processes in the zebrafish will have a bearing on our understanding of the molecular mechanisms underlying distinct human corneal diseases.

A crucial issue for the exploitation of the virtues of the zebrafish in analysis of the cornea is the knowledge of the stage when the cornea has developed to a mature state. It was previously reported that the overall layering of the cornea is evident by 5 to 6 days after fertilisation [7,15]. At this state the three cellular layers of epithelium, stroma and endothelium have formed and have been separated by Bowman’s layer and Descemet’s membrane, respectively. Indeed, our comparative gene expression analysis including the 7 dpf cornea as well as that of the juvenile (1-month) and adult zebrafish (older than 3-months) suggested that most genes expressed in the adult cornea have started expression already at 7 dpf. This indicates that the cornea has matured with respect to its function by 7 dpf. This is paralleled by the onset of hunting that requires visual perception of the prey at the same early larval stage. There are, however, exceptions such as the *frzb* gene encoding a regulator of Wnt signalling. *frzb* mRNA is highly restricted to the ventral iridocorneal angle of the anterior chamber at 7 dpf (Fig. 4L). By 1 month of age, however, *frzb* mRNA expression has spread along the entire endothelial layer.

Many of the cornea genes are also expressed in the retina. Studies of the corneal transcriptome in other model species have frequently relied on subtractive approaches between cornea and other eye structures [48]. Our strategy to analyze cornea expression relative to the skin transcriptome thus reveals a different set of genes. Onset of expression in the retina seems to be delayed with respect to corneal expression of the genes. This suggests that expression of the corneal genes is somehow linked to later maturation or maintenance mechanisms of the retina. In this context it may be of relevance that quite a number of the corneal genes are expressed in the ciliary marginal zone, the retinal stem cell niche, which assures a lifelong growth of the zebrafish retina [27,28].

### Mutation of *pax6b* causes anterior chamber dysgenesis and corneal defects

*pax6b* mutants show dysgenesis of the anterior chamber including a reduced volume of the anterior chamber, lens defects and an incomplete separation of the cornea and the lens. Specifically, a uniform and coherent endothelial layer of the cornea fails to form and also the corneal epithelial layer shows defects. These phenotypic changes bear strong resemblance to the morphological hallmarks of Peters anomaly [3].

In addition to these morphological phenotypes, homozygous *pax6b* mutants are characterised by reduced gene expression in the epithelium and an ectopic expression of genes in the corneal endothelium. The majority of tested corneal genes were affected by the *pax6b* mutation, demonstrating a central role of *pax6b* in corneal gene expression. The affected genes included transcriptional regulatory genes such as *foxc1, pitx2, sox3, foxq1, ctnnb2* and *tfap2a*. This suggests that the affected genes may not all be direct targets of *pax6b*. Based on correlation analysis of *PAX6* mutations and clinical phenotypes, human PAX6 was proposed as a hub gene that modifies other disease-related specifier genes [49]. The central role of *pax6b* in coordinating gene expression in the zebrafish cornea is in line with this proposition of *pax6* being a hub gene.

During eye development, *pax6b* is expressed at low levels in mesenchymal cells presumably representing migrating neural crest cell derivatives taking up their position at the inner surface of the nascent cornea. Later, in the morphologically matured cornea, *pax6b* is much more strongly expressed in the epithelium. This *pax6b* expression correlates well with the observed loss of gene expression in the epithelium of the cornea in *pax6b* mutants. The observed ectopic gene expression in the incorrectly differentiated endothelium may thus be an indirect effect and may indicate cross-talk between the corneal layers. Alternatively, it could reflect the earlier, low expression of *pax6b* in mesenchymal cells or other parts of the nascent eye. The observation in periocular mesenchymal cell culture experiments that *pitx2* and *foxc1* have to become down-regulated for endothelium development to complete may be of importance in this context [50].

The teleost genome was duplicated at the base of vertebrate radiation. Over further evolution many duplicated genes were lost or the duplicated genes were sub-functionalized, taking over specific sub-functions of the ancient unduplicated genes [51]. Thus, rather than being refractory to gene function analysis, this ancient duplication event offers great opportunities to study specific aspects of gene functions. Zebrafish encode two Pax6 genes, *pax6a* and *pax6b*. The phenotype of *pax6b* homozygous mutants is much less severe than that of a homozygous mutation of *Pax6* in mouse [4]. In fact, homozygous *pax6b* zebrafish mutants resemble phenotypically heterozygous Pax6 mutant mice and humans.

## Conclusion

In summary, the zebrafish cornea resembles the human cornea very much, by morphology as well as by gene expression. Moreover, homozygous *pax6b* mutants share striking phenotypic similarities to human patients heterozygous for *PAX6* mutations. The cornea develops fast in the zebrafish embryo and zebrafish *pax6b* mutants are viable with full fertility. The *pax6b* mutant zebrafish will thus represent a promising model for screening of drugs suppressing the development of Peters anomaly and other phenotypes of anterior chamber dysgenesis.

## Materials and Methods

### Ethics Statement

All zebrafish husbandry and experimental procedures were performed in accordance with the German animal protection regulations and were approved by the Regierungspräsidium Karlsruhe, Germany (Aktenzeichen 35–9185.64).

### Fish stocks

Zebrafish (*Danio rerio*) AB_2_O_2_ wildtype strain and *sunrise* Pax6b mutant line were used in this study. Fish were maintained at 28°C as previously described [52].

### Microarray

For each biological repeat of the microarray analysis, 30 eye globes were removed from the sockets and the cornea was carefully isolated with forceps in phosphate-buffered saline. The dermis of the skin was prepared from ten fish by scraping off the scales that hold the epidermis on their outer surface. A scalpel was kept at the right angle against the body surface and scales were completely removed by moving it in a tail to head direction. Inevitably, small amounts of fin and muscle tissues contaminated the dermis preparation. The epidermis of the skin was obtained as described above. Tissues were immediately chilled on crashed blocks of dry ice, weighed and homogenized in TRIzol (Invitrogen) with a blade-type mechanical homogenizer.

To compensate for the different incorporation efficiencies of the dyes, cRNA probes from the cornea and the skin were labelled either with Cy3 or Cy5 dyes in all four possible combinations (“Dye-swap”). Reverse transcription reactions of the isolated total RNA followed by cRNA probe synthesis were conducted with the Low RNA Input Linear Amplification kit (Cat. No. 51843523, Agilent Technologies). Synthesized cRNA probes were purified by QIAgen RNeasy purification kit and quantified by NanoDrop ND-1000 UV-VIS Spectrophotometer (Thermo Fisher Scientific). In total, six G2518A Agilent 22k and two G2519F 4x44k zebrafish microarray chips were used to analyse three to four independent biological repeats. Hybridisation to the microarray was carried out at 65°C for 17 hours, followed by stringency washes and scanning using the Axon model 4000B dual-laser scanner and the corresponding GenePix6 software (Molecular Devices, Union City, CA). Two emission channels for detection of Cy3 and Cy5 dyes, respectively, were scanned in parallel (16-bit colour depth).

All microarray data from this study is MIAME (Minimum Information About a Microarray Experiment) compliant and has been deposited in NCBI’s (National Center for Biotechnology Information) Gene Expression Omnibus under the accession number GSE24599.

### Probe annotation and data processing

Because different Agilent microarray designs were employed in this study only the probes found common to both Agilent arrays (11215 probes representing 8767 genes) were considered for further analysis. The probe sequences commonly found on the Agilent G2518A (design ID:013223 Option 001, 60-mer oligos) and G2519F microarray (design ID: 019161-V2) were re-mapped onto the Zebrafish genome Zv8 and Unigene ESTs by an in-house Perl script after either BLAT- or BLAST-sequence alignment [53,54]. 16450 out of 23763 Agilent probes (69.2%) can be re-mapped onto databases of either Unigene (zebrafish build #117), Ensembl genes (Zv8, release 54) or RefSeq genes (zebrafish release 38). BLAT-/BLAST hits within the coding region or within 500 bp range of either 5’- or 3’-flanking region of start or stop codon, respectively, were regarded as a hit (regardless of UTR or not) of the corresponding gene.

The open source package for microarray analysis, Limma [55] was used to process the data in the following steps: background correction with the “minimum" method, within-array normalization, between-array normalization using the “Aquantile" method, application of linear models for analyzing dye-swap and biological repeats, and finally the assessment of differential expression employing empirical Bayesian for multiple comparisons and the TREAT method (*t*-test relative to a threshold, [56]) for selecting probes with biological meaningful difference.

### RT-qPCR

Total RNA from adult zebrafish cornea and skin was prepared as described for the microarray analysis. Three independent biological repeats were analysed. To avoid contamination of genomic DNA, one microgram of total RNA was treated with one unit of DNase I (Fermentas) before cDNA synthesis with RevertAid H Minus M-MuLV Reverse transcriptase following the manufacturer's instructions (Fermentas). Quantitative RT-PCR (RT-qPCR) was performed with the Applied Biosystems StepOnePlus Real-Time PCR Systems, followed by calculation for Ct (cycle threshold) values with the StepOne Software 2.0 (Applied Biosystems). The reaction mix was based on QuantiTect SYBR Green PCR Master Mix (Qiagen) and performed in a total volume of 20 μl per well of 96-well plates. The template cDNAs were diluted appropriately to obtain a Ct value of 20.7 ±0.4 with the primer pair for the zebrafish *ef1a* gene (ENSDART00000023156.3). All primers (S2 Table), which were designed with the Universal ProbeLibrary web application (Roche Applied Science), were used at the final concentration of 1 μM. Data were analysed by using the 2-^ΔΔCt^-method as described [57]. Expression levels were normalized with the *ef1α* signal as suggested [58]. The statistical significance of the difference in the means of expression levels was determined with the unpaired two-tail *t*-test.

### Cluster analysis

Cluster analysis was performed by an open source software Cluster 3.0 [59] and visualized by Java Treeview [60]. For each of the chosen two sets of data (for example, genes and their expression levels), the similarity of two elements was measured by Kendall’s tau metric and hierarchically clustered by the average linkage method.

### *In situ* hybridisation

For embryonic stages up to 30 hpf, whole mount *in situ* hybridisation was conducted as described [61]. In short, embryos were fixed with 4% (w/v) paraformaldehyde (PFA) in phosphate-buffered saline (PBS) overnight at 4°C. To increase the penetrance of the probes and antibodies, embryos were soaked into -20°C acetone for 7 minutes. Embryos were further processed for hybridisation with digoxigenin (DIG)-labelled antisense riboprobes (see S3 Table for probe information), followed by a sequence of stringency washes, incubation with anti-DIG Fab fragments conjugated with alkaline phosphatase (1:4000 dilution, Roche), and colour development with bromo-4-chloro-3-indolyl phosphate (BCIP, Roche) and nitroblue tetrazolium chloride (NBT, Roche). For reasons of comparison, wildtype and mutant embryos were stained always in parallel and the colour reaction was terminated at the same time. After colour development, the embryos were dehydrated through a series of increasing concentrations of ethanol (50%, 70%, 95%, 100% (v/v)), followed by 100% propylene oxide and a series of increasing concentrations of EPON 812 (glycid ether 100, Serva) in propylene oxide (30%, 70%, 100% EPON/ propylene oxide mixture). Polymerisation was done at 65°C in the presence of 20.8% (w/w) DDSA (dodecenylsuccinic acid anhydride, Serva) and 23.3% (w/w) MNA (methylenacid anhydride, Serva), with the accelerator 1.8% (w/w) DMP 30 (2,4,6-tris(dimethyl-aminomethyl)phenol, Serva). The polymerised block was trimmed and 5 μm semi-thin sections were cut with glass knives using an RM2065 microtome (Leica Microsystems).

For *in situ* hybridization to sections of the adult eye, the eye was removed from the fish sacrificed by application of tricaine methanesulfonate (MS-222). The lens was immediately removed by a small incision where the optic nerve enters the eye. After fixation in freshly prepared 4% (w/v) PFA in PBS at 4°C overnight, tissues were cryo-protected in 30% (w/v) sucrose/PBS and embedded into Jung tissue freezing medium (Leica Microsystems). Sections of 12–14 μm thickness were cut on a cryostat (Leica Microsystems) and recovered on SuperFrost Ultra Plus glass slides (Menzel-Gläser), followed by drying on a heat block at 50–55°C at least for 30 minutes. Sections were kept at -20°C until *in situ* hybridisation. For *in situ* hybridisation, slides with sections were briefly re-dried on a heat block at 50–55°C at least for 30 minutes and washed in PBS. After post-fixation with 4% PFA for 20 minutes, samples were washed with PBS and incubated in the hybridisation buffer (2xSSC, 50% (v/v) formamide, yeast tRNA (10 mg/ml), heparin (50 mg/ml), 0.1% (v/v) Tween20, and 2 mM 2, 2’, 2”, 2”‘-(ethane-1,2-diyldinitrilo)tetraacetic acid (EDTA)) at 65°C for 3 hours (pre-hybridisation). After pre-hybridisation, hybridization was conducted at 65°C for 16 hours with hybridisation buffer including DIG-labelled RNA probes (1:200 dilution, 200 μl scale). Each slide was covered with a cover glass and placed in a plastic container with tissue papers soaked with water to avoid drying. Stringency washes were conducted with 50% formamide/1xSSC at 65°C, three times for 30 minutes each. Samples were incubated briefly with MABT buffer (100 mM Maleic acid, 150 mM NaCl, 0.1% (v/v) Tween20, pH 7.5) and further incubated in a blocking buffer (1% (w/v) Blocking Reagent (Cat. No. 11096176001, Roche) in MABT) for 1 hour. After the blocking procedure, the same protocol was used as described earlier for embryonic stage *in situ* hybridisation.

### Transmission electron microscopy

Embryos were fixed at 7 dpf with Karnovsky fixative, 2.5% (w/v) glutaraldehyde, 2% (w/v) paraformaldehyde, 0.1 M PIPES pH 7.0, overnight at 4°C. Embryos were briefly washed with 0.1 M PIPES pH 7.0 and treated with secondary fixative for 1 hour at 4°C in 0.8% (w/v) K_3_[Fe(CN)_6_], 0.5% (w/v) OsO_4,_ 0.1 M PIPES pH 7.0. The samples were contrasted with 2% (w/v) uranylacetate in 25% (v/v) ethanol at 4°C for several days. After gradual dehydration through an ethanol series (25%, 50%, 70%, 90%, 95% and 100%) and 1,2-propylene oxide, the samples were embedded into EPON 812 resin, as described earlier. Ultrathin sections (“silver” 70 nm thick) were prepared with a Leica EM UC6 ultramicrotome and sections were recovered on formvar-coated slot grids. Samples were examined with a Zeiss T109 transmission electron microscope with the magnification of x7000.

## Supporting Information

S1 Table A list of differentially expressed genes between the cornea and the skin. This table summarizes the genes identified to be differentially expressed between the cornea and the skin of adult zebrafish (adjusted *p*-value <0.001). Genes were sorted into six groups (groups A, B, C, E, F, G, as indicated in the scheme inside) after two rounds of microarray experiments (each with three biological repeats), comparing the cornea against the dermis and epidermis, respectively. The group A corresponds to the “cornea” genes described in the manuscript (260 genes).
(XLSX)

S2 Table Primers used for RT-qPCR. (XLSX)

S3 Table Probe information for *in situ* hybridisation. DIG-labelled antisense riboprobes for cornea genes were prepared from template DNAs derived either from plasmid vector clones (IMAGE consortium clones [62] or own cDNA archives [63]) or PCR products. In the latter case, a set of primer sequences and the source of template DNAs (genomic DNA or cDNA from adult corneae) are given.
(XLSX)

S1 Archives Micrographs of gene expression for selected 72 genes. An archives file (ZIP format) that contains 72 files (PDF format), each corresponding to one of the examined “cornea” genes. Each micrograph has a self-explanatory caption, a sequence of metadata information separated by underscores: (gene symbol)_(stage)_(genotype)_(probe orientation)_(objective lens magnification)_(other additional notes for internal use). 1mpf: 1 month post fertilisation. wt: wildtype. AS-probe: antisense probe for the detection of protein coding transcripts. S-probe: sense probe for the detection of transcripts from the complementary noncoding strand.
(ZIP)
