# Supplementary material for: Molecular Description of Eye Defects in the Zebrafish Pax6b Mutant, sunrise, Reveals a Pax6b-Dependent Genetic Network in the Developing Anterior Chamber
Source: PLoS One. 2015 Feb 18;10(2):e0117645. doi: 10.1371/journal.pone.0117645 (PMC4334901; doi:10.1371/journal.pone.0117645)
Supplement: S1 Archives — An archives file (ZIP format) that contains 72 files (PDF format), each corresponding to one of the examined “cornea” genes. Each micrograph has a self-explanatory caption, a sequence of metadata information separated by underscores: (gene symbol)_(stage)_(genotype)_(probe orientation)_(objective lens magnification)_(other additional notes for internal use). 1mpf: 1 month post fertilisation. wt: wildtype. AS-probe: antisense probe for the detection of protein coding transcripts. S-probe: sense probe for the detection of transcripts from the complementary noncoding strand. (ZIP) [file pone.0117645.s004.zip › arf5.pdf]

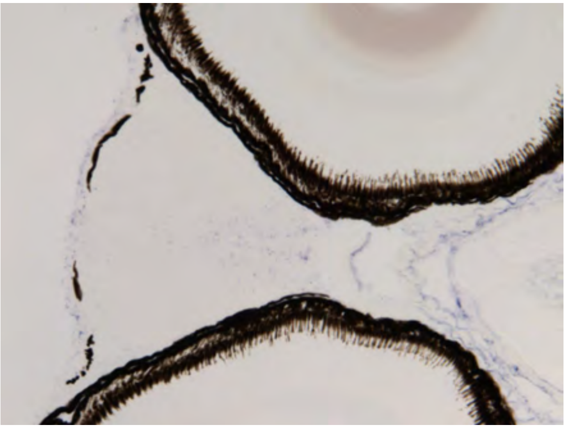

arf5_1mpf_wt_AS-probe_x20_brain_arf5

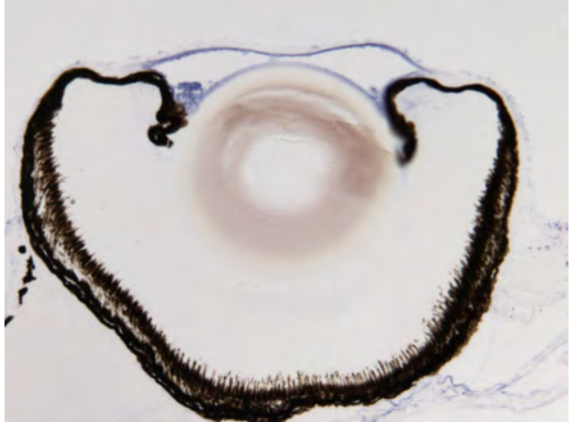

arf5_1mpf_wt_AS-probe_x20_eye_arf5

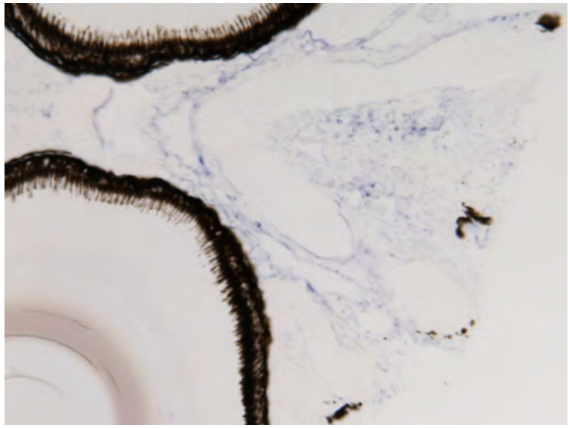

arf5_1mpf_wt_AS-probe_x20_pharynx_arf5

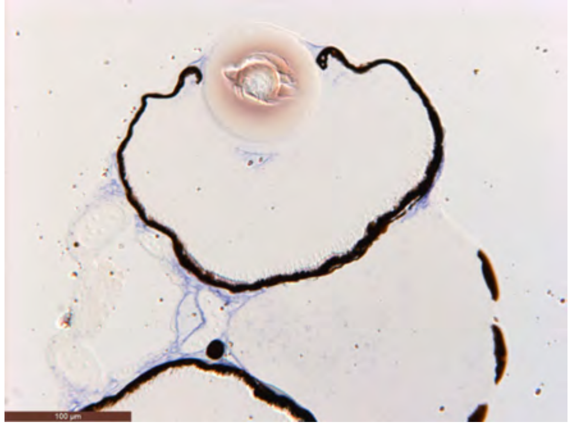

arf5_7dpf_pax6b-mutant_AS-probe_x20_arf5_0

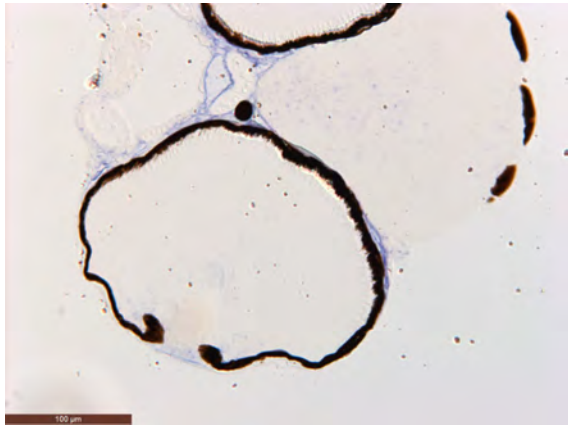

arf5_7dpf_pax6b-mutant_AS-probe_x20_arf5_01

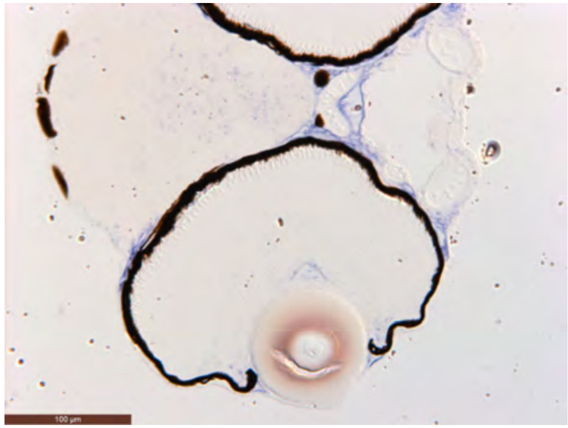

arf5_7dpf_pax6b-mutant_AS-probe_x20_arf5_02

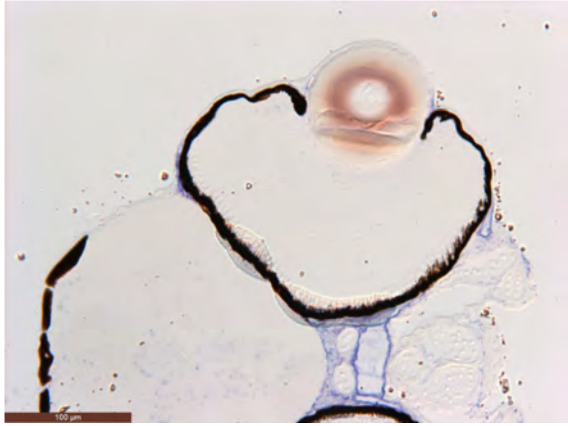

arf5_7dpf_pax6b-mutant_AS-probe_x20_arf5_03

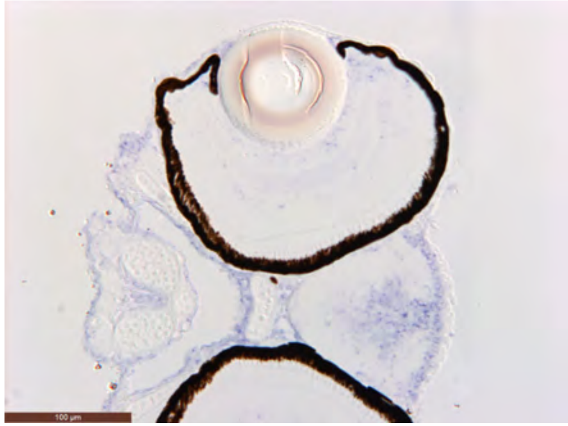

arf5_7dpf_wt_AS-probe_x20_arf5_0

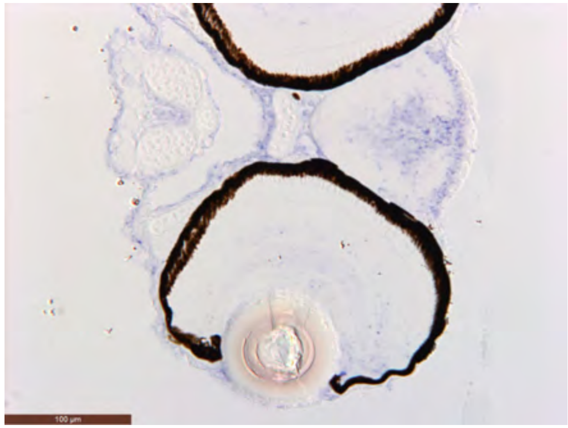

arf5_7dpf_wt_AS-probe_x20_arf5_01

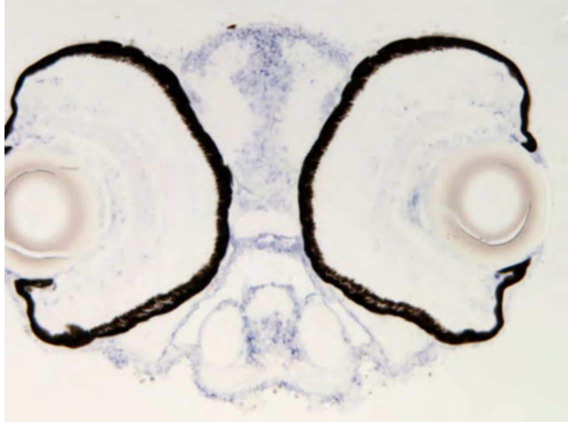

arf5_7dpf_wt_AS-probe_x20_arf5

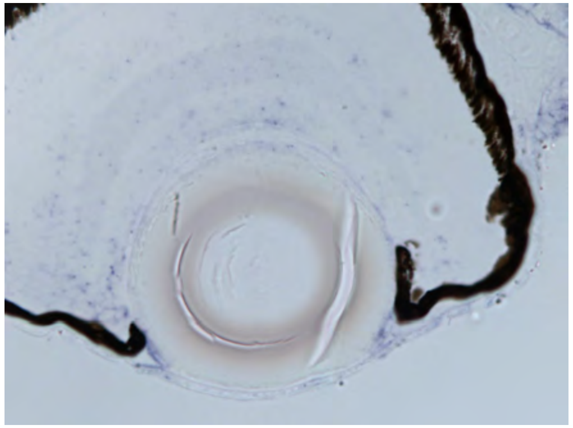

arf5_7dpf_wt_AS-probe_x40_arf5_02

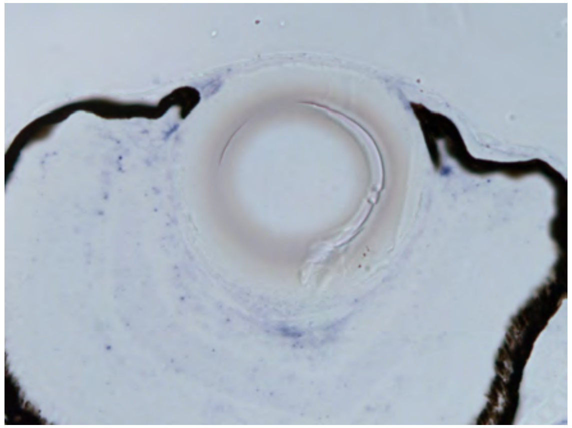

arf5_7dpf_wt_AS-probe_x40_arf5
